# Targeting New Candidate Genes by Small Molecules Approaching Neurodegenerative Diseases

**DOI:** 10.3390/ijms17010026

**Published:** 2015-12-25

**Authors:** Hueng-Chuen Fan, Ching-Shiang Chi, Shin-Nan Cheng, Hsiu-Fen Lee, Jeng-Dau Tsai, Shinn-Zong Lin, Horng-Jyh Harn

**Affiliations:** 1 Department of Pediatrics, Tung’s Taichung Metroharbor Hospital, Wuchi, Taichung 435, Taiwan; 2 Department of Nursing, Jen-Teh Junior College of Medicine, Nursing and Management, Miaoli 356, Taiwan; fanhuengchuen@yahoo.com.tw (H.-C.F.); chi-cs@hotmail.com (C.-S.C.); pedcsn@yahoo.com.tw (S.-N.C.); 3 Department of Pediatrics, Taichung Veterans General Hospital, Taichung 407, Taiwan; leehf@hotmail.com; 4 School of Medicine, Chung Shan Medical University, Taichung 402, Taiwan; Fernand.tsai@msa.hinet.net; 5 Department of Pediatrics, Chung Shan Medical University Hospital, Taichung 402, Taiwan; 6 Graduate Institute of Immunology, China Medical University, Taichung 404, Taiwan; shinnzong@yahoo.com.tw; 7 Center for Neuropsychiatry, China Medical University and Hospital, Taichung 404, Taiwan; 8 Department of Neurosurgery, China Medical University Beigang Hospital, Yunlin 651, Taiwan; 9 Department of Pathology, China Medical University and Hospital, Taichung 404, Taiwan

**Keywords:** neurodegenerative diseases (NDs), Parkinson’s disease (PD), Huntington’s disease (HD), Alzheimer’s disease (AD), epigenetics, Ataxia-telangiectasia-mutated protein kinase (ATM), neuroinflammation

## Abstract

Neurodegenerative diseases (NDs) are among the most feared of the disorders that afflict humankind for the lack of specific diagnostic tests and effective treatments. Understanding the molecular, cellular, biochemical changes of NDs may hold therapeutic promise against debilitating central nerve system (CNS) disorders. In the present review, we summarized the clinical presentations and biology backgrounds of NDs, including Parkinson’s disease (PD), Huntington’s disease (HD), and Alzheimer’s disease (AD) and explored the role of molecular mechanisms, including dys-regulation of epigenetic control mechanisms, Ataxia-telangiectasia-mutated protein kinase (ATM), and neuroinflammation in the pathogenesis of NDs. Targeting these mechanisms may hold therapeutic promise against these devastating diseases.

## 1. Introduction

Neurodegeneration occurs when structures or functions of neurons are progressively lost. Acute neurodegenerative diseases (NDs), such as stroke [1] or traumatic brain injury [2] may result neurons partial or entire loss; chronic NDs, including Parkinson’s disease (PD) [3], Huntington’s disease (HD) [4], Alzheimer’s disease (AD) [5], *etc.*, are among the most feared of the disorders that afflict humankind because of the lack of specific diagnostic tests and effective treatments. The World Health Organization (WHO) predicts that NDs are going to overtake cancer in the rank of top causes of death by 2050 [6]. The estimated figures, severity and chronicity of these diseases, and a vast economic and emotional burden on individuals, communities and governments generate an urgent need to better understand pathophysiology, improve early diagnosis and develop effective treatments of NDs.

Early studies suggested that misfolding proteins or polyglutamine-dependent pathogenesis resulted in an excessive amount or abnormal structural aggregation-prone proteins accumulation, causing several distinct NDs [7]. However, numerous theories, such as impaired ubiquitin-proteasome and/or autophagy-lysosomal pathways [8], mitochondrial dys-function [9], programmed cell death [10], glutamatergic activity, reactive oxygen species (ROS), *etc.*, suggest the complexity of NDs. Insights into the cellular and molecular pathogenesis of NDs may broaden our understanding of the underlying mechanisms and hold therapeutic promise against debilitating CNS disorders. Hence, this review will discuss the clinical manifestations of three distinct NDs: PD, HD, and AD through the associated molecular machineries, including epigenetic misregulation, Ataxia-telangiectasia-mutated protein kinase (ATM), and neuroinflammation. Greater understanding of the diseases may aid development of better therapeutics.

## 2. Molecular Mechanisms of NDs

### 2.1. Epigenetic Misregulation

**Figure 1:**
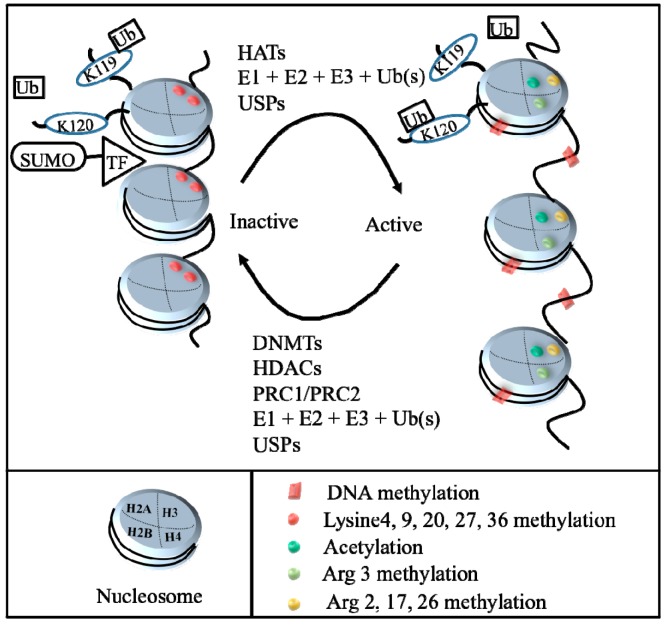
Illustration of epigenetic mechanisms. The process of DNA condensation and relaxation is controlled principally through histone post-translational modifications, such as methylation, acetylation, phosphorylation, ubiquitination, sumoylation, *etc*. Histone acetyltransferases (HATs), mono- and poly-ubiquitination, and ubiquitin-specific proteases (USPs) may cause uncoiling chromatin (euchromatin) and allow transcriptional factor access to the DNA (right); whereas DNA methyltransferases (DNMTs), histone deacetylases (HDACs), mono- and poly-ubiquitination (Ub), ubiquitin-specific proteases (USPs), Polycomb repressive complex 1(PRC1)/Polycomb repressive complex 2 (PRC2), and Small ubiquitin-related modifier (SUMO) modification may result in coiling chromatin (heterochromatin) and prevent transcription factor access to DNA, leading to transcriptional repression. TF: transcription factor.

Normally, DNA is tightly coiled into dense chromatin and not available for reading and transcription in the nucleus of mammals. Relaxation of chromatin allows DNA active transcription. Nucleosomes, the smallest structural unit of chromatin, are composed of eight histone core molecules, including doublets of histone 2A, 2B, 3, and 4, with two loops of 147 bp DNA. The process of coiling and uncoiling the genome is mainly modulated via post-translational modifications. Although there may appear to be a bewildering array of histone modifications, there are at least methylation, acetylation, phosphorylation, ubiquitination, sumoylation, *etc.* [11]. These epigenetic changes involve the covalent chemical reactions of histones by DNA methyltransferases (DNMTs), histone acetyltransferases (HATs) and histone deacetylases (HDACs), the polycomb repressive complex 1 (PRC1) and PRC2, ubiquitination- and sumoylation-related proteins to regulate activation or inactivation of gene expression (Figure 1).

DNMTs contain at least four subtypes, including DNMT1, DNMT2, DNMT3a and DNMT3b. After DNA replication, the parent strand preserves methylated but the newly synthesized strand dose not. DNMT1 binds to these hemi-methylated CpG sites and methylates the cytosine on the newly synthesized stand to maintain established CpG methylation patterns through mitosis. DNMT2 may have a very low DNA-cysteine methylation activity though DNMT2 may possess a protective function [12]. DNMT3a and DNMT3b are essential for methylation and early development, and the loss of either is lethal [11].

HATs adopted an acetyl group from acetyl-coenzyme A to counterbalance the positive charge of the lysine residues on the N-terminal tails of H2A, H2B, H3 and H4 [13] to uncoil chromatin for transcriptional factors (TF)s to access and initiate transcription. This reaction is reversed by HDACs, which take away the acetyl groups from the lysine to coil chromatin and inhibit TF approaching. *HDAC* isoforms are extensively expressed in the brain and are at least 18 isoforms, which have been characterized and phylogenetically categorized into four main classes: Class I HDACs include HDACs 1, 2, 3, and 8. Class II HDACs is divided into class IIa, consisting of HDACs 4, 5, 7 and 9, and class IIb consisting of HDACs 6 and 10. Class III, the NAD^+^ dependent class, comprises of Sirtuin 1, 2, 3, 4, 5, 6, and 7. Class IV consists of HDAC 11 [14]. HDACI is multifunctional, including abolishing aberrant epigenetic modifications and abnormal transcriptional imbalance, modulating cytoskeletal and immune functions, and enhancing protein degradation. Pharmacological interventions using HDAC inhibitors (HDACI) are promising in the treatment of several diseases, including cancers, metabolic diseases, neuropsychiatric diseases, and NDs [15,16,17].

Methylation is one of modifications of histone to regulate transcriptional expression and orchestrate numerous genes. Methylation on arginine or lysine residues of H3 or H4 can trigger a transcriptional cascades [18]. DNMTs transfer a methyl group, which is from *S*-adenosyl methionine (SAM), to target molecules. Moreover, most CpG islands are associated with functional genes and may contain promoters. Methylated CpG islands or gene promoter regions may cause gene silencing or activation. CpG methylation may partially rely on the ratio between SAM and *S*-adenosyl-homocysteine (SAH) [19]. After methylation, the methyl group is then back to SAH after cleavage by histone demethylases (HDMs). Furthermore, multiple methylation valences, including mono-methylated, di-methylated or trimethylated lysine and arginine residues on H3 and H4 are noted. For instance, activation signals include methylation at H3-Lys(K)4, K36, K79; Arg2, Arg17, Arg26 and H4-Arg3, usually link to uncoiling chromatin structure; inhibition signals include H3-Lys9, K27, K36, and H4-K20, Arg8 and Arg3 [20,21].

There are at least two subtypes of Polycomb (PcG) proteins: PRC1, and PRC2. PRC1, which catalyzes the mono-ubiquitination of histone H2A and plays a role in silencing maintenance, contains Bmi1/MEL18, polyhomeotic (PH), Ring1a, Ring1b and CBX/HPC PcG proteins. PRC2 is associated with transcriptional repression. PRC2 is composed of embryonic ectoderm development (EED), suppressor of zeste 12 (Suz12), zeste homolog 2 (EZH2) and RBAP48/46. PRC2 initially attached to chromatin and EZH2 and trimethylated H3K27 [22]. PRC1 recognized the methylated markers. E3 adhered RING1/2 and then mono-ubiquitinated H2AK119, making chromatin coiled and causing transcriptional repression [23]. EZH2 trimethylated H3K27, causing repression of promoters [24]. Excessive-expression EZH2 inhibits BRCA1 phosphorylation and thereby facilitates cell proliferation in breast cancer [25]. The histone methylation inhibitor 3-deazaneplanocin A (DZNep), a well-known EZH2 inhibitor, is promising in the treatment of cancers and other diseases [26].

Ubiquitin is a highly conserved protein. Four genes, including UBB, UBC, UBA52 and RPS27A produce ubiquitin in the human genome [27]. Ubiquitination, is an enzymatic process, containing ubiquitin-activating enzymes (E1), which can activate the ubiquitin; ubiquitin conjugating enzymes (E2), which is a linker between the ubiquitin and E1; ubiquitin ligases (E3), which connects the glycine 76 of the ubiquitin to a lysine on the substrate protein through an isopeptide bond. In general, substrates, which were mono-ubiquitinated, represented as a signal carrying specific information. Poly-ubiquitinated substrate were for degradation [28]. H2A and H2B can be mono- and poly-ubiquitinated [29,30]. The same ubiquitin binding to H2A or H2B may cause distinct outcomes, because ubiquitinated histone H2A (uH2A) antagonizes transcription [31]. uH2B activates transcription [32]. The ubiquitination site of H2A is lysine 119 (K119). The site of H2B is K120. The reaction of mono-ubiquitination is reversible by ubiquitin-specific proteases (USPs) [33]. PRC1 is as ubiquitin ligase for H2A; PRC2 installs the H3K27me3 marks for PRC1 to recognize. After being ubiquitinated, uH2A repressed gene expression. Rad6 enhanced mono- and poly-ubiquitination on H2A and H2B [32]. Notably, *in vitro* studies showed that BRCA1 assisted ubiquitination on H2A and H2B, but the interactions *in vivo* remain unclear [34].

Small ubiquitin-related modifier (SUMO) modification (sumoylation) occurs on histones and results in transcriptional repression [35]. All four histones are sumoylated in S. cerevisiae [36], whereas only H4 has been identified to be modified in mammalian cells [37]. H4 can connect E2 and be sumoylated in an E1- and E2-dependent pattern. Moreover, several molecules, including the histone demethylase LSD1, the histone methyltransferase SETDB1, chromatin-associated proteins HP1, L3MBTL1 and L3MBTL2, the nucleosome remodeling ATPase Mi-2, and deacetylase HDAC2 were recruited when SUMO proteins were covalently attached to a histone, leading to gene silencing through modulating the chromatin structure dynamics [37]. Because the structure of SUMO proteins is similar to that of ubiquitin [38], their functions may also share similarities. Additionally, sumoylation can affect the distribution of proteins, initiate functions of enzymes, degrade or preserve target proteins, repress transcriptional factors, *etc.* [39].

### 2.2. Ataxia-Telangiectasia and Ataxia-Telangiectasia Mutation (ATM)

**Figure 2:**
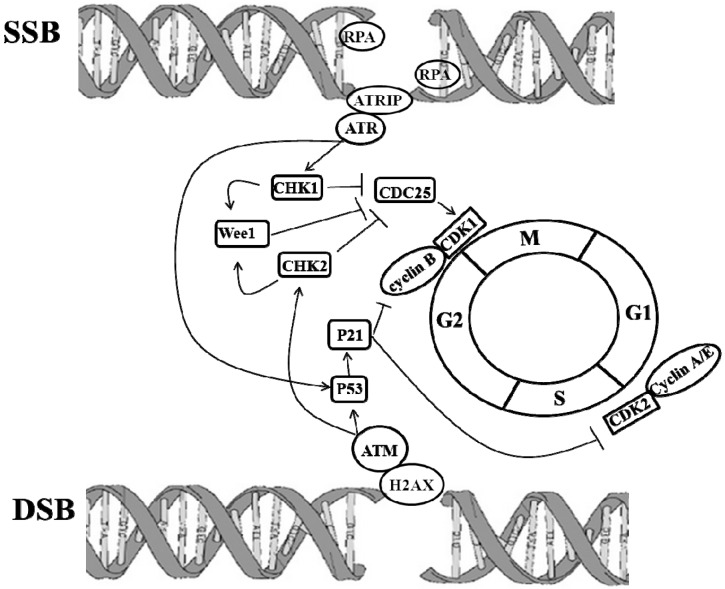
Molecular cascades of DNA damages initiating repair systems and cell cycle progression. In response to DNA damage, including single-strand beaks (SSBs) and double-stranded breaks (DSB), the Ataxia-telangiectasia-mutated protein kinase (ATM)/ Ataxia-telangiectasia and Rad3 related protein (ATR) signaling pathways are activated, leading to the phosphorylation and activation of CHK1 and CHK2 and to the subsequent phosphorylation of CDC25. Phosphorylated CDC25 inhibits activation of cyclin B/CDK1, resulting in G2 arrest. Activated ATM/ATR pathways also activate p53-dependent signaling to arrest G2 through the activation of P21, which inhibits cyclin B/CDK1 complexes. Lines with arrow heads indicate activation, while lines with bar heads indicate inhibition.

Ataxia-telangiectasia (AT), also called Louis-Bar syndrome, is a rare and inherited human disease. A-T is characterized by predisposition to cancer, immunodeficiency and a significant loss of neurons causing neurological conditions [40,41]. The mutated ATM gene produced A-T phenotypes. ATM is a member of the PI3-kinase family and ubiquitously expressed throughout development. ATM involves the DNA repair system and maintains the integrity of its genome by controlling cell cycle checkpoints. When DNA is damaged by UV light, ionizing radiation, or ROS to cause lesions including DNA hydrolysis, DNA oxidation, DNA single-strand beaks (SSBs), and other damages [42]. If damaged DNA is left unrepaired, irreparable and toxic DNA double-strand breaks (DSBs) may be produced [43]. Functionally, ATM is activated by DSBs [44]. At very early step, activated ATM by DSBs can immediately phosphorylate histone H2AX at the site of the break [45]. ATM and Rad3 related (ATR) mutually works with ATR-interacting protein (ATRIP) to recognize SSB, which is fastened by replication protein A (RPA) binding. In response to DNA damage, ATR and ATM stimulated checkpoint kinases CHK1 and CHK2, respectively via P53 dependent and independent signaling pathways [46]. p53 dependent pathways: phosphorylated p53 initiated p21, which inhibited CDK1/cyclin B to regulate cell cycle [47]. p53 independent pathway: CHK1 and CHK2 phosphorylated CDC25, which then down-regulated CDC25A/B/C activity [48], leading to the inhibition of CDK1/cyclin B [49]. CHK1 and CHK2 activated Wee 1 through phosphorylation. Phosphorylated CDC25 and Wee 1 arrested cell cycle at G2/M phase [50]. ATM and ATR sense and transduce damaged DNA signals to initiate DNA repair, apoptosis, rest and repair [51,52,53] (Figure 2). For example, BRCA1 and P53, well-known tumor suppression genes (TSG), are regulated by ATM [54]. Mutated BRCA1 and P53 may be involved in the development and progression of cancers, and in the pathogenesis of NDs. Moreover, a study showed that ATM-mediated phosphorylation of EZH2 reduced protein stability [55,56]. Knockdown EZH2 improved the histological degeneration of Purkinje cells and mitigated behavioral impairment in ATM KO mice [57], suggesting that epigenetics modulate ATM mediated NDs.

### 2.3. Neuroinflammation: Pro-Inflammatory and Anti-Inflammatory Cytokines, and Irregular Tryptophan (TRP) Metabolism

#### 2.3.1. Pro-Inflammatory and Anti-Inflammatory Cytokines

Neuroinflammation was originally defined as inflammation of the CNS, which may be triggered by viral and bacterial infection, ischemic stroke, toxic metabolites, HIV encephalopathy, and autoimmunity [58,59]. Immune responses can be triggered by these causes and exposed self-antigens in damaged CNS, causing autoimmune reactions that commonly follow these curs, because circulating peripheral immune cells can surpass a compromised blood-brain barrier (BBB) and activate the immune response to protect the CNS. Also, microglia, the resident innate immune cells in the CNS, act as scavengers to eradicate microbial pathogens, modulate immune responses and generate neurotrophic or toxic substances to trigger diseases [60]. The CNS is typically an immunologically privileged site because peripheral immune cells are blocked by the BBB. However, the widespread inflammation in the CNS attracted further migration of leukocytes infiltration, leakage, and disruption of the BBB, leading to neurodegeneration [61].

TNF and the IL family are pro-inflammatory cytokines. They attract leucocytes and amplify proliferation at the inflammation site, synthesize proteolytic enzymes, initiate cytotoxicity, and secrete other pro-inflammatory factors to continue inflammation. These cytokines stimulated IL-6 production to generate anti-inflammatory cytokines and other immune factors to neutralize pro-inflammatory effects [62]. Imbalance between pro-inflammatory and anti-inflammatory cytokines may possibly cause neuroinflammation and NDs. For example, formation of α-synuclein (SNCA) fibrils were aggregated in PD in neuroinflammation [63]. Elevated circulating inflammatory cytokines and monocytes with hyper-responsive to immune stimuli were found in HD patients and HD mouse models [64]. Moreover, several studies discovered excessive pro-inflammatory cytokines in microglia, astrocytes, and neurons, and co-localization with both Aβ plaques and tau, and increasing Aβ and tau phosphorylation in the AD’s brain [65]. Apart from traditional viewpoints regarding the inflammation, the field of neuroinflammation has broadened to enroll NDs, such as PD, HD, and AD. These diseases lack the signs of classic “inflammation”, such as immune cell infiltration from the blood stream, but they featured cellular and molecular icons of neuroinflammation, including imbalance of pro-inflammatory and anti-inflammatory cytokines expression, microglia activation, *etc*.

#### 2.3.2. Irregular Tryptophan (TRP) Metabolism

TRP is the precursor of two important metabolic pathways, serotonin synthesis and kynurenine (KYN) synthesis. It is estimated that 95% of mammalian serotonin is found within the gastrointestinal tract [66], and only about 1% of dietary TRP is converted to serotonin in the brain [67]. The serotonin pathway generates melatonin, which may affect neural and endocrine systems that regulate circadian rhythms of behavior, physiology, and sleep patterns [68].

**Figure 3:**
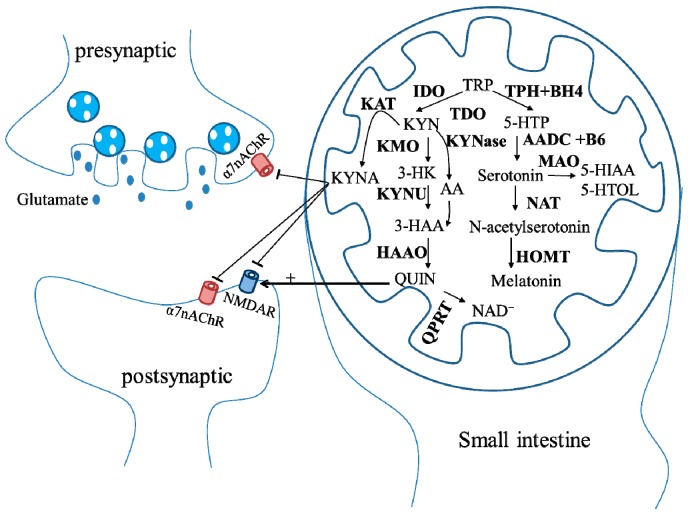
Schematic representation of the tryptophan (TRP) metabolic pathway. Most TRP is used as the precursor of kynurenine (KYN) pathway, in which TRP is firstly oxidized by TRP 2,3-dioxygenase (TDO) or indoleamine 2,3-dioxygenase (IDO) to the kynurenine (KYN). There are at least three pathways for KYN metabolism. (1) KYN aminotransferase (KAT) pathway: KYN is catabolized by KAT to form KYN acid (KYNA), which antagonizes *N*-methyl-D-aspartate receptors (NMDAR) and α7 nicotinic receptors (α7nAChR); (2) kynurenine 3-monoxygenase (KMO) pathway: KYN is converted into 3-HK by KMO. 3-HK is converted into 3-HAA by KAT; (3) KYNase pathway: KYN is metabolized by KYNase to form anthranilic acid (AA), which is converted into 3-hydroxyanthranilic acid (3-HAA) by AA3MO. 3-HAA is oxidized by 3-hydroxyanthranilic acid oxidase (HAAO) to quinolinic acid (QUIN), which generates NAD^+^ through quinolinate phosphoribosyltransferase (QPRT). Additionally, TRP is metabolized to 5-hydroxytryptophan (5-HTP) through TRP hydroxylase (TPH) and tetrahydrobiopterin (BH4). Serotonin is synthesized from 5-HTP via aromatic acid decarboxylase (AADC) and the vitamin B6. Serotonin is converted into 5-Hydroxyindoleacetic acid (5-HIAA) or 5-hydroxytryptophol (5-HTOL) by monoamine oxidase (MAO) or into *N*-acetylserotinin by N-acetyl-transferase (NAT). Melatonin is generated via the hydroxyl-indole O methyltransferase (HOMT).

The KYN pathway accounts for approximately 90% of TRP catabolism [69,70]. TRP is firstly oxidized by TRP 2,3-dioxygenase (TDO) or indoleamine 2,3-dioxygenase (IDO) to KYN. There are at least 3 pathways for KYN metabolism: (1) KYN aminotransferase (KAT) pathway: KYN is catabolized by KAT to form KYN acid (KYNA); (2) kynurenine 3-monoxygenase (KMO) pathway: KYN is converted into 3-hydroxykynurenine (3-HK) by KMO. 3-HK is converted into 3-hydroxyanthranilic acid (3-HAA) by KAT; (3) KYNase pathway: KYN is metabolized by kynureninase (KYNase) to form anthranilic acid (AA), which is converted into 3-HAA by anthranilate 3-monooxygenase (AA3MO). 3-HAA is oxidized by 3-hydroxyanthranilic acid oxidase (HAAO) to quinolinic acid (QUIN), which generates NAD^+^ through quinolinate phosphoribosyltransferase (QPRT) [71,72]. Additionally, TRP is metabolized to 5-hydroxytryptophan (5-HTP) through TRP hydroxylase (TPH) and tetrahydrobiopterin (BH4). Serotonin is synthesized from 5-HTP via the aromatic acid decarboxylase (AADC) and the vitamin B6. Serotonin is converted into 5-Hydroxyindoleacetic acid (5-HIAA) or 5-hydroxytryptophol (5-HTOL) by monoamine oxidase (MAO), or into *N*-acetylserotinin by *N*-acetyl-transferase (NAT). Melatonin is generated via the hydroxyl-indole O methyltransferase (HOMT) (Figure 3).

KYN pathway plays a crucial role in the neuroinflammation because this pathway is affected by several pro-inflammatory cytokines, which may interfere with enzyme expressions. Consequently, excessive pro-inflammatory cytokines favor the KMO branch of the pathway [72]. In addition to affecting dopamine, norepinephrine, β-endorphin, serotonin and endocrine, such as cortisol, prolactin and growth hormone, TRP also initiates excitotoxicity through the generation of KYNA to inhibit *N*-methyl-D-aspartate (NMDA) receptors [73] and α7 nicotinic receptors [74], and the generation of QUIN to activate *N*-methyl-D-aspartate (NMDA) receptors [75], all linking to the pathogenesis of NDs (Figure 3). Derangements of the TRP metabolism may possibly directly or indirectly lead to accumulation of neurotoxicity, causing PD, HD, or AD.

## 3. Distinct NDs Share Common Molecular Mechanisms

### 3.1. Parkinson’s Disease (PD)

PD is apparently motor neuron degenerative disorder because it affects total 0.3% of the population in industrialized countries, more than 1% of 60-year old individuals, and 4% of those ages over 80 [76]. Investigating the brain in a PD patient showed progressive degeneration and loss of dopaminergic (DA) neurons and degenerative nerve terminals in the basal ganglion [77], resulting in typical PD presentations, including tremor, rigidity, and hypokinesia [77]. Before the onset of symptoms, at least 70% of neurons exhibit degenerative dys-function or neuronal loss in PD patients [78]. Although most PD are sporadic; however, some PD are familial and affects young patients. Familial PD inherited models can be divided into an autosomal dominant (AD) or autosomal recessive (AR) pattern. It has been reported that at least 13 loci and 9 genes link to PD. Six genes are connected to Mendelian patterns of PD. SNCA and leucine-rich repeat kinase 2 (LRRK2) are associated with ADPD, while Parkin, phosphatase and tensin homolog (PTEN)-induced kinase 1 (PINK1), DJ-1, and ATPase type 13A2 (ATP13A2) are linked to ARPD [3]. The main pathological features of these degenerative neurons in PD is SNCA protein, which aggregates to form Lewy bodies and Lewy neuritis to cause numerous detrimental consequences on neurons [79]. In addition to genes and related products, aberrant protein folding, degeneration, impaired DNA repair, abnormal cell cycle, excessive mitochondria and oxidative stress, and uncontrolled inflammation and microglial activation, synaptic malfunction, and dys-functional ubiquitin proteasome system are all implicated in the pathogenesis of PD [80]. Complicated pathogenesis and disease progression suffered PD patients and confused clinicians. Medical treatments such as dopamine replacement therapies may produce unwanted effects. As surgical intervention only can partially control symptoms, a deeper understanding of the underlying mechanisms of PD may direct better therapies.

#### 3.1.1. PD and Epigenetic Misregulation

Demethylation of a CpG rich island in SNCA was found in patients with PD, resulting in increased expression of SNCA *in vitro* [81]. Accumulated experiences and knowledge from *in vitro* and *in vivo* experiments and trials, Kontopoulous *et al.* [82] suggested a sequence regarding the effect of the H3 hypoacetylation: preventing histone acetylation, condensing chromatin, repressing gene expression and ultimately leading to cell death, suggesting the therapeutic potential of HDAC manipulation. In agreement with these suggestions, consistent reports showed that pretreat VPA or TSA could resist inflammation, which was induced by lipopolysaccharide (LPS), and protect DA neurons [83]. TSA, VPA, and Class I HDAC-specific inhibitors apicidin and MS-275 could protect neurons against inflammation [84]. Phenylbutyrate prevented the loss of DA cells through up-regulated the DJ-1 protein [85] and reduction the level of phosphorylated SNCA [86]. Although VPA was effective in several *in vitro* and *in vivo* experiments, two studies failed to prove the effects of VPA on disease progression in PD patients [87,88].

#### 3.1.2. PD and ATM

ATM is possibly involved in the pathogenesis of PD because (1) extrapyramidal presentations, such as chorea or dystonia, may be associated with A-T [89,90,91,92]; (2) ATM gene knockout (ATM KO) mice exhibited severe loss of tyrosine hydroxylase-positive DA nigro-striatal neurons, and midbrain DA neurons initially developed normal axons to the striatum, but degenerated later in life [93]; (3) SNCA was detectable in the cortex, striatum and substantia nigra (SN) of ATM KO mice [93]; (4) In cancers, *PARK2* and *ATM* mutations sometimes occur synchronically at the same amino-acid residue, causing neuronal degeneration [94,95,96]. This overlap suggests that cancers and PD may adopt similar mechanisms. Any known therapeutic approaches to one of these two distinct disorders may also be a novel approach to the other disorder.

#### 3.1.3. PD and Neuroinflammation

Several studies detected that the numbers of microglia expressing major histocompatibility complex (MHC) class II molecules in the SN and putamen were frequently correlated with SNCA-positive Lewy neurites and monoaminergic neuritis, and the numbers of MHC class II-positive microglia were increased when the degeneration of the SN proceeded [97,98]. IL-6 and TNF-α were detected in the reactive microglia in the CNS of PD [98]. Levels of pro-inflammatory cytokines, including IL-1β, IL-2, IL-4, IL-6, TNF-α and TGF-α were elevated in the cerebral spinal fluid (CSF) and basal ganglion of PD brain [99,100]. High level of pro-inflammatory cytokines may dys-regulate the KYN pathway in PD. KAT in the substantia nigra was significantly decreased when MPTP (1-methyl-4-phenyl-1,2,3,6-tetrahydropyridine) or 6-OHDA (6-hydroxydopamine) injected into mice [101,102]. Treatment with MPP^+^ (1-methyl-4-phenylpyridinium ion) diminished KAT and KYNA concentration in the CNS of rats [103]. Decreased KYNA levels and increased 3-HK levels were found in PD post-mortem brain [104], and increased IDO activity was detected in serum and CSF samples of PD when compared to healthy subjects [105]. KYNA can attenuate the MPP^+^ related neurotoxicity in human neuroblastoma cells [106]. Injected into animals with substrate for KYNA, l-KYN can improve signs and symptoms of PD induced by 6-OHDA [107]. QUIN reduced striatal tyrosine hydroxylase activity [108], which can be reduced by KYNA injection into the rat’s brain [109]. Also, in the MPTP mice model, synthetic KYNA analogs may have neuroprotective properties against PD through targeting mitochondria [110]. Together, KYNA or its analogs may have neuroprotective effects in PD. Additionally, administrated Ro 61-6048, a KMO inhibitor, alleviated dystonia in an animal model [111,112], and reduced the levodopa-induced dyskinesia in PD animal model [113,114]. The presence of activated microglia, excessive pro-inflammatory cytokines and dys-regulated KYN pathway suggested that neuroinflammation is crucial in the underlying mechanisms of PD.

### 3.2. Huntington’s Disease (HD)

HD is one of the most common types of PolyQ diseases with a frequency approximately a 1%–3% new mutation rate and 10 cases per 100,000 per year [115]. Clinical presentations are variable, including initially cognition or mood subtle changes to later poor or complete loss of coordination, an unsteady gait, jerky body movements, a prominent decline in mental and behavioral abilities, and dementia. Patients with HD normally die within 20 years of symptom onset [116]. Typical neuropathological features of HD include atrophy of the basal ganglion with significant gliosis and neuronal loss. Huntingtin (HTT) expressed in neurons of the CNS with unknown functions. However, mutant HTT (mtHTT) and its proteolytic fragments may cause neurons more susceptible to aging stresses and death [117]. The expansion of CAG repeats within the HTT protein may damage mitochondria, chaperones, and the ubiquitin proteasome system in the neurons, leading to neurodegenerative phenotypes [118,119,120]. There is no effective treatment for HD.

#### 3.2.1. HD and Epigenetic Misregulation

Hypo-acetylation and hypermethylation were detected in patients, animal models, and cell line models of HD [121,122]. Impairment of hippocampal neurogenesis, which was related to DNA methylation, might possibly link to cognitive decline in HD [123]. Levels of hypo-acetylation of histone were associated with down-regulated genes in HD, and HDACI revitalized the hypo-acetylation and improved the motor signs/symptoms of HD [122,124,125,126,127]. The H3K9me3 associated heterochromatin might be a pathological feature of HD [128]. The stretching CAG repeats in mtHTT interacted with CBP to cause neurons of HD transcriptional dys-function [129,130]. Butyrate, one of HDACI, improved motor performance and neuropathologic sequelae, enhanced memory, reduced polyglutamine-induced toxicity, and significantly extended survival of HD mice [131,132,133,134]. However, a multi-center, placebo-controlled double-blind investigation did not support the use of phenylbutyrate in HD patients [135,136]. A study using lithium and VPA co-treatment alleviated locomotor deficits and depressive-like behaviors in HD mice [137]. TSA was also shown to suppress polyglutamine toxicity in Caenorhabditis elegans [138]. Pimelic diphenylamide, one of HDACI 4b, can modulate gene networks, inhibit kappa B kinase, and eradicate the HTT protein [127]. Suberoylanilide hydroxamic acid (SAHA), a potent HDAC inhibitor, significantly improved motor symptoms of HD mice [139]. AGK2, a SIRT 2 (sirtuin 2) inhibitor, was shown to diminish mutant huntingtin toxicity in cellular and invertebrate models of HD [140]. Anthracyclines, including mithramycin and chromomycin A, both of which are DNA-binding drugs against the rapid growth of tumors [141]. Mithramycin ameliorated the neuropathological phenotypes and extended survival through reducing pericentromeric heterochromatin condensation in the HD mice model [122,134]. Chromomycin re-built the balance of acetylation and methylation of histone H3K9, and improved phenotypes of HD mice [142]. Mithramycin and Chromomycin can partially restore the perturbed histone modifications, suggesting a potential therapeutic function. However, mithramycin and chromomycin have not been used in humans because of their toxicity.

#### 3.2.2. HD and ATM

ATM signaling is elevated in HD cell [143,144], mouse [144] models, as well as the fibroblasts from HD patients [145]. ATM KO significantly reduced toxicities in animal cells and in transgenic insect models with mutant Huntingtin (mHTT) fragments [143]. Genetic reduction using cross-mating with mices carrying heterozygous ATM allele and other mices expressing full-length human mHTT, showed significant improvement in several behavioral deficits and partial improvement in neuropathology [143]. KU-60019, a small-molecule ATM inhibitor, can rescue death of rat striatal neurons expressing mHTT and iPS cells from HD patients [143]. Together, the reduction of ATM signaling may ameliorate mHTT toxicity in cellular and animal models of HD.

#### 3.2.3. HD and Neuroinflammation

TRP was negatively correlated with symptom severity and number of CAG repeats [75,146,147]. QUIN and KYNA, metabolites of TRP, may trigger excitotoxic neuronal death in HD through the modulation of NMDAR and α7nAChR [148,149]. The administration of QUIN in the basal ganglions generated cellular, neurochemical and behavioral changes similar to human HD [75,147]. 3-HK, another metabolite of TRP, is also involved in the pathogenesis of HD [150]. Elevated levels of 3-HK were detected in the brain of a HD mice model [151]. Damage of mutant HTT expressing neurons initiated microglia activation, causing excessive cytokines production and IDO transcription, and overflow of unwanted neuroactive KYN metabolites [152]. Levels of 3-HK and QUIN were higher in the brain and striatal neurodegeneration of Transgenic mice expressing the full-length mutant HTT proteins [148]. Direct injections with QUIN into the mice’s striatum produce histological, biochemical, and neuropathological changes resembling those observed in human HD [153]. Elevated levels of 3-HK and QUIN were detected in the post-mortem HD brain [148]. Genetic deletion of KMO in yeast cells expressing mtHTT protein attenuated CAG repeats-mediated toxicity and decreased 3HK and QUIN [154]. In Drosophila expressing mtHTT protein, pharmacological or genetic inhibitions of KMO ameliorated the loss of neurons [155]. In the HD mouse model, inhibition of KMO preserved synapses, mitigated microglia activation, and improved survival [156]; SzR-72 [*N*-(2-*N*,*N*-dimethylaminoethyl)-4-oxo-1*H*-quinoline2-carboxamide hydrochloride], a KYNA analog, was reported to improve hypolocomotion, increase weight, prevent striatal neurons atrophy, and increase survival [157]. Moreover, Ro61-8048 (3,4-dimethoxy-*N*-[4-(3-nitrophenyl)thiazol-2-yl]benzenesulfonamide), an analog of the KMO inhibitor, was found to suppress mtHTT-mediated toxicity [154]. Therefore, degrees of inherited genetic abnormality and clinical disease severity may drive the way of TRP metabolism along the KYN pathway in HD [75,147]. Inhibiting KMO and/or amplifying KYNA may possess neuroprotective effects in HD.

### 3.3. Alzheimer’s Disease (AD)

Patients with AD may typically present debilitating progressive neurodegeneration and manifest memory, cognitive and behavioral impairment, and dementia. Statistical data show that there will be 115.4 million individuals worldwide expecting to have AD by the year 2050 [158], and the medical care for AD would likely be worth over US$600 billion per annum in the USA [159]. The prevalence of AD is more common in people at ages of 60s, and early onset of AD may occur in people at ages of 40s and 50s [160]. Central pathological hallmarks in the brain of a patient with AD are (i) genes; (ii) amyloid plaques; (iii) neurofibrillary tangles, which is hyperphosphorylated microtubule-associated protein tau (MAPT) and other pathological changes associated with this neurodegenerative disorder; (iv) inflammation; (v) gliosis; (vi) oxidative stress; (vii) neuronal dystrophy; (viii) neuronal loss; (ix) synapse loss; (x) altered levels of neurotransmitter; and (xi) cell cycle [161]. Clinically these pathological changes are correlated with symptoms and sign like the progressive deficit in memory, predominantly short-term memory loss in early stages [162]. As AD progresses, cognitive function worsens, the central pathological hallmarks become prominent and brain atrophy gets momentum [163,164]. Although increasing tau proteins were detected in the brain of AD patients than healthy individuals [165], however, significant amyloid deposition is detected in the brain of elderly subjects without cognitive impairment [165]. The solution for these contradictory data is pending.

#### 3.3.1. AD and Epigenetic Misregulation

Folate and vitamins B12 and B6 are potentially protective effects to counteract cognitive decline and possibly the onset of dementia. Vitamin B was shown to delay the progression of dementia in AD [166], and *in vivo* showed reduction in DNA methylation in AD patients [167,168]. Vitamin B deficiency induced hypomethylation of CpG sites near the Presenilin 1 (PS1) promoter [169]. *In vitro* folate deprivation induced global DNA hypomethylation, leading to an increased expression of PS1 [170]. SAM supplementation successfully restored the folate deficiency-induced abnormalities [171]. Additionally, levels of folate and SAM in CSF, and levels of SAM in the brain cortex, were found to be decreased in AD cases [172], concomitant with an increase in brain SAH levels [173]. Deprived folate, vitamin B12 and vitamin B6 in mutant human APP transgenic mice showed increased SAH to SAM ratios and increased PS1 levels [174]. Tau gene expression is involving differentially methylated binding sites for transcription factors [175,176]. Disturbed cell-cycle control and subsequent induction of apoptosis in AD neurons were related to DNA methylation [177,178,179]. Histone acetylation was found to be drastically decreased in the temporal lobe of AD patients [180], and in animal models of AD [181]. KO HDAC2 was shown to ameliorate the cognitive problems and aberrant synaptic plasticity [181]. It was reported that levels of H3 phosphorylation was increased in the frontal cortex of AD patients [182], but levels of H3 methylation was decreased in the striatum [183]. The AD mice were found to show increased H3 acetylation levels were found in the prefrontal cortex, and increased H4 acetylation levels in the CA1 region of the hippocampus [183]. HDAC6 levels were found to be significantly higher in the hippocampus of AD [184]. HDAC6 may tau phosphorylation and aggregation [185] through deacetylation [186], or deacetylate HSP90 to affect tau clearance [186]. Reduction of HDAC6 levels mitigated learning and memory problems in an AD mouse model [187]. SIRT1 was found to be up-regulated in AD mouse models [188], but to be decreased in AD patients [189]. Additionally, SIRT1 may deacetylate tau [189]. MS-275, an HDACI, was able to partially alleviate behavioral deficits, neuroinflammation and plaque load in AD mice [180]. Decreased acetylation of H4 was found to link to memory impairments in the AD animal model, and administration of TSA could alleviate symptoms [190]; Administration of VPA could modify synapses and accelerate neurite outgrowth via the GSK 3βpathway [191]. However, apart from hypomethylation, a differential cortex-specific hypermethylated region of ankyrin 1 (ANK1) was found to be associated with the early stages and the progression of AD [192]. Therefore, DNA hypo/hypermethylation may facilitate cell death.

#### 3.3.2. AD and ATM

The expression of ATM was significantly elevated in the cerebellar dentate neurons of AD patient [193]. Aberrant persistence of H2AX, a marker of ATM activation, was found in the brain of a familial AD mouse model [194]. ATM deficient neurons re-entered the cell cycle and died [195,196], suggesting that ATM may protect neuron by stopping cells re-entering the cell cycle and lessening DNA damage. Moreover, p53 and BRCA1 may possibly play a role in the pathogenesis of AD because both of them triggered apoptosis in neuronal cells [197]. p21 and p27 are tumor suppressor proteins and are stimulated by BRCA1. It was reported that p21 and p27 are linking to the development of AD [163,198], suggesting that tumor suppressor molecules may manipulate AD progression. Also, ATM impairment or deficiency in glial cells may trigger innate immune responses to cause NDs [199]. The histology of microglial cell in ATM KO mice was abnormal, and astrocytes from ATM KO mice showed significant expressions of oxidative and endoplasmic reticulum stress and a senescence-like reaction [200,201]. ATM deficiency may disturb DNA repair, trigger apoptosis, and accelerate aging and neuroinflammation.

#### 3.3.3. AD and Neuroinflammation

Altered immune responses have been observed in AD [202,203]. Excessive pro-inflammatory cytokines, including IL-1β, IL-6, IL-8, TNF-α, IFN-γ, and KYN, and lower levels of TRP were detected in serum and brain samples of AD patients [204,205,206]. Elevated Aβ1-40 and Aβ1-42 in transgenic AD mice were associated with increased TNF-α, IL-6, and IL-1β [207]. Microglia, which were cultured from AD patients, generated excessive TNF-α, pro-IL-1β, IL-6, IL-8, macrophage colony stimulating factor (M-CSF), and complement proteins, especially C1q, when they were stimulated by Aβ1-42 [208]. When β-APP is engulfed and digested by the microglia, a surge of IL-1, IL-6, TNF-α, ROS, NO, proteolytic enzymes, KYN metabolizing enzymes and complement proteins, was consequently released by chronically excited microglia in the AD patients [209]. Aβ1-42 human activated IDO expression in microglia and monocytes [207,210]. Astrocytes, oligodendrocytes, and endothelial and neuronal cells were involved in neuroinflammation, because the expressions of IDO and QUIN were augmented in microglia, astrocytes, and neurons of AD patients [210]. Coptisine, the IDO inhibitor, could ameliorate cognitive impairment in an AD mouse model [211]. The detrimental cytokine-mediated induction of KYN metabolism may contribute to either independently or in combination with amyloid or other factors to cause synaptic dys-function and neuronal loss, leading to the development of AD [212].

## 4. Conclusions

Since the WHO predicts that NDs will surpass cancer in the rank of top 10 cause of death by 2050, any effective therapy for NDs is needed. Potential drugs or interventions for the treatment of NDs are summarized in the Table 1. A spate of new therapeutics targeting HDAC for treating various types of disorders, such as diabetes, systemic lupus erythematosus (SLE), hepatocellular carcinomas, leukemia and lymphoma, include siRNA HDAC [213], SAHA [139], PCI-24781 (Abexinostat) [214], ITF-2357 (Givinostat) [215]; MS-275 (Entinostat) [216], MGCD 0103 (Mocetinostat) [217], LBH-589 (Panobinostat) [218], FK228 (Romidepsin) [219], AGK2 [220], and PXD-101 (Belinostat) [15]. These novel chemicals have been under investigation as monotherapy or in combinatorial therapy as alternatives or adjuvants to traditional therapies. Nevertheless, epigenetic misregulation, ATM, and neuroinflammation may individually or symphonically involve the pathogenesis of NDs. The use of HDACI may potentially be able to rescue the deteriorated functions of NDs. Genetic ablation and pharmacological inhibitors of ATM can reduce mt-HTT toxicity to protect neurons. Amplifying KYNA and/or mitigating 3-HK and QUIN, and the use of KMO inhibitor reduced the dystonia and dyskinesia and improved striatal dys-functions through the attenuation of neuroinflammation. Synchronically targeting the three mechanisms may not only preserve neuron cells, inhibit cell death, and limit neuroinflammation, leading to slowing or alleviating the symptoms of PD, HD, and AD, but also offer potential beneficial therapy for other neurocognitive disorders in the future.

**Table 1:** Potential drugs or interventions for the treatment of neurodegenerative diseases (NDs), including Parkinson’s disease (PD), Huntington’s disease (HD), and Alzheimer’s disease (AD), are classified by mechanisms, including epigenetic misregulation, ATM, and neuroinflammation.

Mechanisms	Epigenetic Misregulation	Reference	ATM	Reference	Neuroinflammation	Reference
NDs						
PD	VPA	[87,88]	ATM KO	[93]	l-KYN	[107]
	TSA	[83,84]			Ro 61-6048	[111,112]
	Butyrate	[85,86]				
	MS-275 (Entinostat)	[211]				
	AGK2	[216]				
HD	VPA	[137]	KU-60019	[143]	SzR-72	[157]
	TSA	[138]			Ro 61-8648	[155]
	Butyrate	[131,132,133,134]				
	Pimelic diphenylamide	[127]				
	SAHA	[139]				
	AGK2	[140]				
AD	VPA	[191]			Coptisine	[211]
	TSA	[190]				
	MS-275 (Entinostat)	[189]				
